# Use of Experimental and Numerical Results in the Prototyping of 3D-Printed Water Jets

**DOI:** 10.3390/ma18184367

**Published:** 2025-09-18

**Authors:** Paweł Madejski, Dominik Buksa, Mateusz Bryk

**Affiliations:** 1 Department of Power Systems and Environmental Protection Facilities, Faculty of Mechanical Engineering and Robotics, AGH University of Krakow, Adama Mickiewicza 30, 30-059 Krakow, Poland; 2 Strata Mechanics Research Institute, Polish Academy of Sciences, Reymonta 27, 30-059 Krakow, Poland; 3 Energy Conversion Department, Institute of Fluid-Flow Machinery, Polish Academy of Sciences, Fiszera 14, 80-231 Gdansk, Poland; mbryk@imp.gda.pl

**Keywords:** water nozzle, fused deposition modeling, rapid prototyping, computational fluid dynamics

## Abstract

Rapid prototyping methods have gained increasing interest across various industries in recent years. Among these methods, 3D printing technology is increasingly used for prototype production, mainly due to its relatively low development and manufacturing costs and the short time required for physical fabrication. This paper presents the integration of 3D printing methods with experimental results as an example of the application of rapid prototyping techniques to examining water jet nozzles. Four different nozzle shapes were investigated. Water nozzle design enables control over the flow stream, its range, and efficiency, tailored to specific requirements. Computational Fluid Dynamics (CFD) was combined with 3D printing to ensure fast answers and to enable a comparison of the proposed solutions. Nozzle prototypes were manufactured using an FDM-based 3D printer and PLA material. The new evaluation criteria coefficient is proposed to calculate the nozzle printing fabrication efficiency.

## 1. Introduction

Additive manufacturing is a production technology that creates three-dimensional physical objects by applying successive layers of material until the designed shape is achieved. Three-dimensional printing allows such objects to be printed directly from a computer-aided design (CAD) model [1]. This method has emerged as cost-effective, as it involves less material consumption, lower human involvement, and reduced energy consumption during production compared to traditional manufacturing processes [2]. Three-dimensional printing technologies are divided into seven main categories according to the ISO/ASTM standard, namely Binder Jetting (CJP), Directed Energy Deposition (LENS), Material Extrusion (FDM), Material Jetting (Polyjet), Powder Bed Fusion (SLS/SLM), Sheet Lamination (LOM), and Vat Photopolymerization (SLA/DLP) [3]. Three-dimensional printing is gaining increasingly widespread use, spanning various fields such as surgery, the pharmaceutical industry, implant manufacture, prosthetics, and tissue engineering [4]. It allows for increasingly advanced use, including the personalization of medicine by customizing dosages and pharmaceutical forms to individual patients’ needs, as well as the creation of multi-ingredient tablets and controlled-release forms of active ingredients [5]. The dynamic development of additive methods has also led to the emergence of bioprinting, an advanced form of 3D printing that involves the layering of cells and biomaterials. This technology enables the creation of tissue structures and could be used to create full-sized organs in the future, thereby opening up new possibilities in the fields of regenerative medicine and transplantology [6]. Its versatility extends further to the energy industry, enabling sustainable production by reducing material wastage and CO_2_ emissions. Three-dimensional printing is used, among other uses, to manufacture parts for wind turbines and fuel cell components, contributing to the development of renewable energy sources [7]. The use of 3D printing technology in these sectors is of particular interest because the related devices often require the use of expensive, advanced materials, such as ceramics or composites, which are subject to significant limitations in terms of their shape and functionality when using traditional manufacturing methods [8]. In aerospace, 3D printing technology is gaining popularity because it is expected to reduce the weight of components by as much as 50–80%, resulting in direct advantages through reduced fuel consumption and lower carbon dioxide emissions. The manufacturing processes most often use titanium alloys, nickel alloys, and polymer composites reinforced with glass or carbon fibers. An additional advantage is the high recycling potential of the waste generated during printing, estimated at 95–98% [9].

Industrial applications are increasingly combining rapid prototyping technologies with numerical simulation (CFD). One particular development is observed in the design of new aircraft, where CFD models are a crucial tool. The aerospace industry is increasingly combining rapid prototyping technologies with numerical simulations (CFD). The paper [5] describes research on the Generic Future Fighter (GFF) prototype, in which 3D printing enabled the fabrication of wind tunnel models and an actual radio-controlled flying model. The results of the CFD simulations were confirmed by experimental tests, allowing for optimization of the wing design used in the real model. The estimated lead time for the entire project—from concept to flight testing—was 6 to 12 months [10]. Three-dimensional printing plays a crucial role in validating CFD simulations and visualizing them, as it enables models with isosurfaces of the temperature distributions or flows in complex structures to be printed [11].

This article focuses on the applications of water nozzles, which are widely used in both industrial and domestic applications. These components play a key role in daily appliances and in complex technological systems. Their design and operation are adapted to the specifics of the process, especially where high-pressure generation is required—including in washing, descaling, and cooling and sprinkler systems [12]. In the power industry, water jets are used in water turbines to control rotor speed [13], in firefighting systems using water mist [14], and in spray cooling and quenching processes [15]. They are also increasingly important in renewable energy sources: in wind turbines, they assist in de-icing the blades, and in photovoltaic systems, they enable gentle and even cleaning of the panels. Additionally, in modern energy storage systems, water nozzles are used to precisely control humidity by producing fine droplets sprayed into the air, which is important for handling lithium materials [16].

## 2. The Flow Characteristics of Nozzles Developed Through the Additive Manufacturing Technique Using FDM

In the context of the research conducted, water nozzles were fabricated using Fused Deposition Modeling (FDM) additive manufacturing technology. Although SLA printing can provide smoother surfaces and even transparent components that facilitate flow visualization, in this study, we deliberately selected the more accessible and low-cost FDM technology to highlight its limitations and potential for rapid prototyping of hydraulic nozzles. The models of the nozzles were printed with PLA material, with the respective parameters of the 3D printing process appropriately selected to achieve the required geometric accuracy (Table 1). The printed components were then experimentally validated in a custom-designed test rig (Section 2), which was targeted at evaluating the flow behavior for different operating conditions. Operating parameters such as the pressure drop and water flow rate were quantified through experiments. Numerical flow models (CFD) were developed to represent the test conditions for the nozzles. The results obtained during the experimental tests were compared with the results of the numerical simulations to evaluate the effectiveness of CFD computational methods in the analysis of this type of system. Compilation of the results made it possible to verify the accuracy of the models and evaluate the usefulness of computer simulations in the design and optimization of FDM-printed nozzles.

### 2.1. Description of the FDM Method

Fused Deposition Modeling (FDM) technology involves layering molten thermoplastic material to produce three-dimensional objects. FDM is among the most widely used 3D printing techniques, mainly due to the low cost of the materials and their wide availability. The process typically utilizes materials such as polylactide (PLA), acrylonitrile-butadiene-styrene (ABS), and high-performance thermoplastics, including Polyether Ether Ketone (PEEK) and Polyether Imide (PEI) [17]. Thanks to digital control over the model’s volume, it is possible to control the dimensions and mass of the print very precisely. Significantly, FDM prints are characterized by high repeatability, which makes it possible to obtain serial elements with identical properties [18]. The diameter of the filaments used in FDM technology is usually 1.75 mm or 3 mm. The quality of the prints produced by FDM is significantly affected by the thickness of a single layer, the pattern and density of the fill, the speed of printing, the orientation of the model during construction, and the temperatures of the extrusion head and the work table [19]. FDM technology, although widely used, is not a perfect solution and has many limitations. Among the main ones are poor mechanical properties due to weak bonding between layers and limited dimensional accuracy. Prints are also characterized by anisotropy, exhibiting lower Z-axis strength. There is also a widespread surface stepping effect, resulting in high surface roughness. In addition, even optimization of the printing parameters does not always eliminate structural defects, such as pores or inaccurate binders [20].

### 2.2. Water Nozzles Developed with Additive Techniques

Among the crucial parameters established for the FDM printing process of the nozzles was the printer nozzle diameter, which was 0.4 mm, allowing the small details of the internal geometry of the channels to be adequately replicated. The thickness of a single layer was established at 0.1 mm to achieve high surface quality and minimize roughness that could adversely affect fluid flow characteristics. The extrusion temperature was 210 °C, which is optimal for PLA material and ensures enough adhesion between layers. The temperature of the working table was 60 °C, thus stabilizing the printing process and minimizing the risk of deformation of the printed elements. The print speed of 30 mm/s allowed for a compromise between the precision of execution and the production time for the models. Additionally, a 100% fill rate was used to ensure the inner nozzle structures were fully tight [21]. All printing parameters are presented in Table 1.

**Figure 1:**
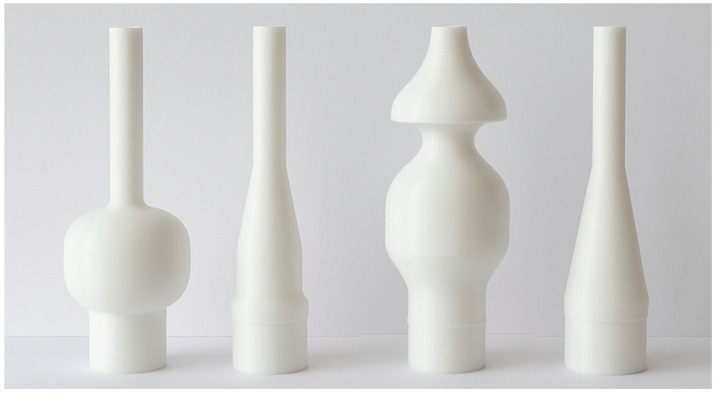
Analyzed nozzles printed using PLA material and the FDM method.

**Figure 2:**
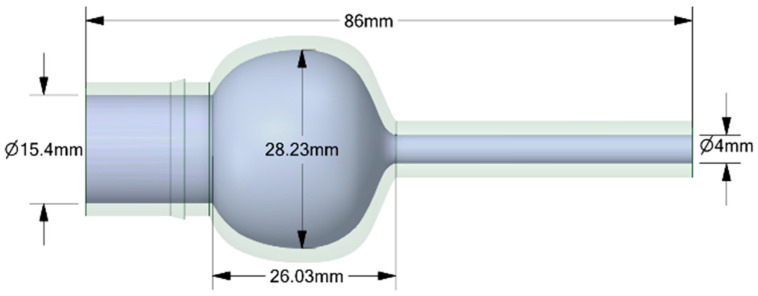
The geometrical model with all dimensions marked for the analyzed nozzle variant D1.

**Figure 3:**
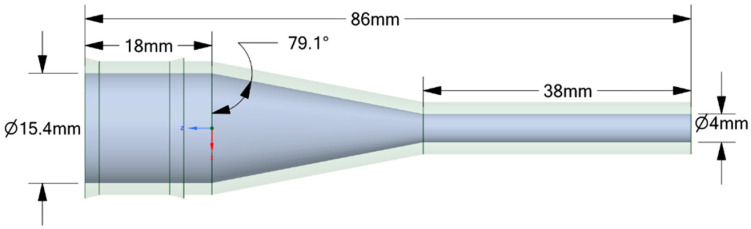
The geometrical model with all dimensions marked for the analyzed nozzle variant D2.

**Figure 4:**
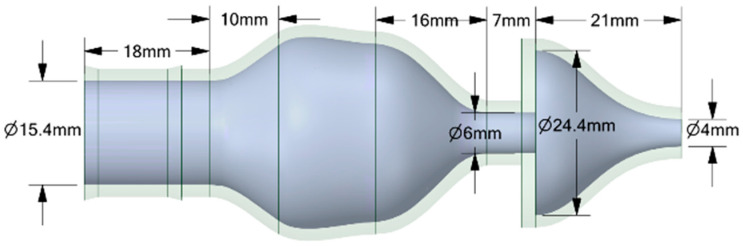
The geometrical model with all dimensions marked for the analyzed nozzle variant D3.

**Figure 5:**
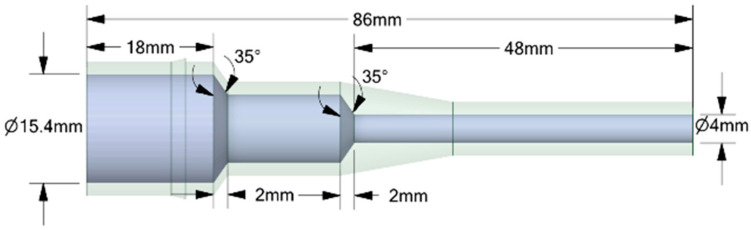
The geometrical model with all dimensions marked for the analyzed nozzle variant D4.

The experiments analyzed four nozzle variants (Figure 1), whose geometry was designed to facilitate a comparison of their flow characteristics (Table 2). The analyzed nozzles (D1–D4) were designed with the same overall length (86 mm), inlet diameter (15.4 mm), and outlet diameter (4 mm). The differences resulted from the internal channel geometry, in particular the convergence angle, the presence of chambers or convergent sections, and the throat length (Figure 2, Figure 3, Figure 4 and Figure 5). According to the technical drawings, the minimum diameter was 4 mm (throat/outlet) and the maximum inlet diameter was 15.4 mm for all variants. Additional chambers enlarged the flow area in some designs; for example, in D1, the intermediate chamber had a diameter of approximately 28.23 mm. The converging sections were designed with an angle of 79.1° in nozzle D2 and 35° in nozzles D4.

## 3. The Measurement System for Experimental Studies of Water Jets

**Figure 6:**
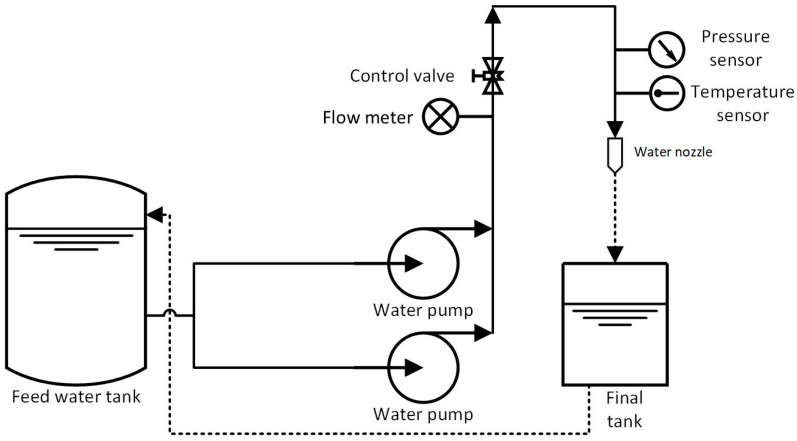
A scheme of the laboratory stand used for experimental investigations of the 3D nozzle prototype.

The test stand was designed with easy connections and water nozzle replacements in mind, allowing for the measurement of the main flow parameters over a wide range of operating conditions, primarily different inlet pressures. A schematic diagram of the stand is shown in Figure 6, and its main components are a feed water tank, two centrifugal pumps (0–16 bar) with an electric motor, inverters, control valves, a mounting system for fixing water nozzles, a final tank, pressure sensors, temperature sensors, and flow meters. The list of sensors, including their range and accuracy, is presented in Table 3.

**Figure 7:**
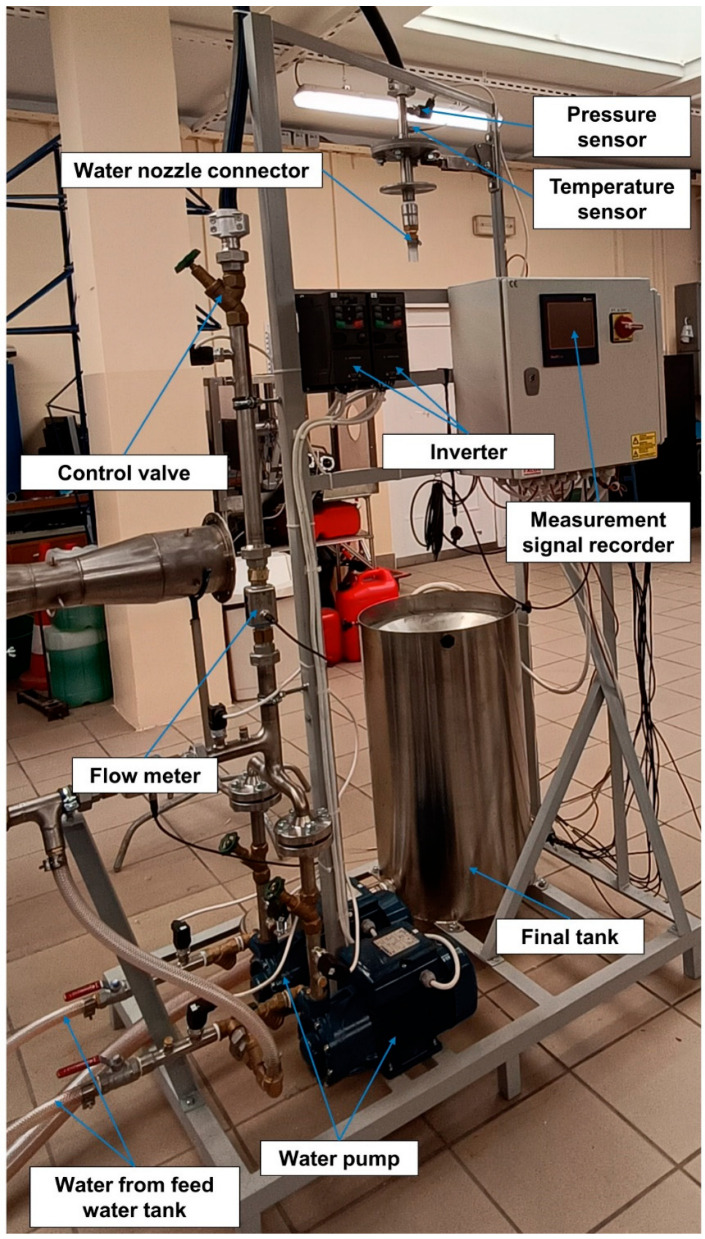
The test stand at the AGH laboratory, featuring marked main components for experimental water jet investigations: feed water tank connections, two centrifugal pumps with electric motors, two inverters, control valves, a mounting system for water nozzle connections, a final tank, pressure sensors, temperature sensors, and flow meters.

The system is controlled by an inverter, which changes the current frequency of the motor powering the centrifugal pumps (Figure 7). This change in frequency results in a change in the rotation of the pump impeller and the resistance posed by the flow system and the nozzle under test. For this purpose, pressure measurements were used at several points in the system, most importantly just before the nozzle under test so that the pressure readings corresponded to the values at the nozzle inlet. In addition to pressure and volume flow rate, temperature measurements were also taken to accurately determine the properties of the water at the actual temperature.

**Figure 8:**
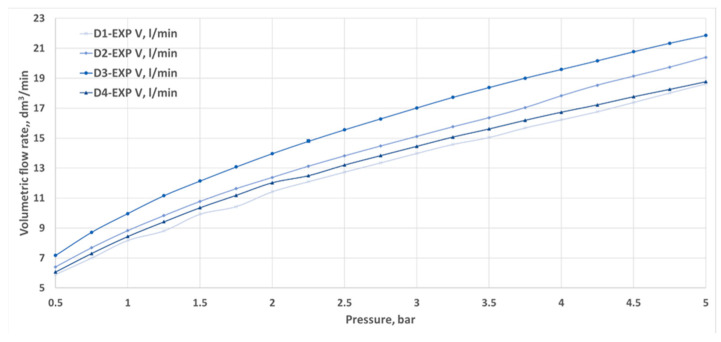
The volumetric flow rate of liquid through the tested water nozzles D1–D4 at different inlet pressures, obtained during experimental tests.

Figure 8 presents the basic results obtained on the test bench, specifically the water flow rate for varying pressures at the inlet for nozzles D1–D4. Changing the parameters through smooth control over the inverter made it possible to set the pressure value in front of the nozzle at the same level, ranging from 0.5 to 5 bar. Pressure changes were made in 0.5 bar increments.

## 4. The Numerical Model of the Water Flow Through a Nozzle

Ansys Fluent was used to solve the governing equations for the mass, momentum, and energy conservation These equations are based on the Reynolds approach to the turbulent flow, from which they were derived as the continuity equation Equation (1) and the conservation of momentum equation Equation (2):

(1) $$\frac{\partial \rho }{\partial t}+\;\frac{\partial }{\partial x_{i}}\left(\rho u_{i}\right)=0$$

(2) $$\frac{\partial }{\partial t}\left(\rho u_{i}\right)+\frac{\partial }{\partial x_{j}}\left(\rho u_{i}u_{j}\right)=-\frac{\partial p_{av}}{\partial x_{i}}+\frac{\partial }{\partial x_{j}}\left[\mu \left(\frac{\partial u_{i}}{\partial x_{j}}+\frac{\partial u_{j}}{\partial x_{i}}-\frac{2}{3}\delta_{ij}\frac{\partial u_{z}}{\partial x_{z}}\right)\right]+\frac{\partial }{\partial x_{j}}\left(-\rho \bar{u_{i}^{\prime }u_{j}^{\prime }}\right)+\rho g$$

where *t*—time, s; ρ—density, kg/m^3^; $u_{i,j,z}$—average velocity magnitude, m/s; $p_{av}$—average pressure, Pa; $x_{i,j,z}$—spatial variables, m; μ—dynamic viscosity, kg/(m s); $\delta_{ij}$—Kronecker delta, -; $\rho \bar{u_{i}^{\prime }u_{j}^{\prime }}$—Reynolds stress tensor, N/m^2^; i,j,z—an indicator of the spatial coordinates, -; g—gravitational acceleration, m/s^2^.

The term $\rho \bar{u_{i}^{\prime }u_{j}^{\prime }}$ in the momentum conservation equation represents the Reynolds stresses associated with turbulence. In this study, the Boussinesq hypothesis was applied to the model, which relates the occurring stresses to the average velocity:

(3) $$\rho \bar{u_{i}^{\prime }u_{j}^{\prime }}=\mu_{t}\left(\frac{\partial u_{i}}{\partial x_{j}}+\frac{\partial u_{j}}{\partial x_{i}}\right)-\frac{2}{3}\left(\rho k+\mu_{t}\frac{\partial u_{z}}{\partial x_{z}}\right)\delta_{ij}$$

where $\mu_{t}$ = turbulent dynamic viscosity, kg/(ms).

Reynolds stresses result from turbulence-induced velocity fluctuations and are not influenced by fluid viscosity. Since they add six unknowns to the Reynolds equations, turbulence models are required to close the system. The energy transport equation used is given as

(4) $$\frac{\partial }{\partial t}\left(\rho E\right)+\frac{\partial }{\partial x_{j}}\left(u_{j}\left(\rho E+p\right)\right)=\frac{\partial }{\partial x_{j}}\left(k_{eff}\frac{\partial T}{\partial x_{j}}+\tau_{ij}u_{i}\right)$$

where $k_{eff}$—effective thermal conductivity, W/(mK); E—internal energy, J/kg; h—enthalpy, J/kg; T—temperature, K; $\tau_{ij}$—the deviatoric stress tensor, Pa.

In the present case, the turbulence effects were modeled using the Shear Stress Transport (SST) k-ω model, which combines the advantages of the standard k-ε and k-ω formulations. This hybrid model transforms the standard k-ε equations into a set of transport equations for the turbulent kinetic energy *k* and the specific dissipation rate ω, where

(5) $$\omega =\frac{\varepsilon }{k}$$

The following equation describes the kinetic energy of turbulence *k*:

(6) $$\frac{\partial }{\partial t}\left(\rho k\right)+\;\frac{\partial }{\partial x_{i}}\left(\rho {ku}_{i}\right)=\frac{\partial }{\partial x_{j}}\left({Ⱶ}_{k}\frac{\partial k}{\partial x_{j}}\right)+G_{k}-Y_{k}+G_{b}$$

while the specific dissipation rate ω

(7) $$\frac{\partial }{\partial t}\left(\rho \omega \right)+\frac{\partial }{\partial x_{i}}\left(\rho {\omega u}_{i}\right)=\frac{\partial }{\partial x_{j}}\left({Ⱶ}_{w}\frac{\partial \omega }{\partial x_{j}}\right)+G_{\omega }-Y_{w}+G_{wb}$$

where *k*—turbulence kinetic energy, J/kg; ω—specific dissipation rate, s^−1^; ${Ⱶ}_{k},{Ⱶ}_{w}-$ effective diffusivity of k and ω, m^2^/s; $G_{k}$—the generation of turbulence kinetic energy due to mean velocity gradients, J/(s m^3^); $G_{\omega }$—generation of ω, kg/(s^2^ m^3^); $Y_{k}$—dissipation of *k*, J/(s m^3^); $Y_{\omega }$—dissipation of ω, kg/(s^2^ m^3^); and $G_{b},G_{wb}$—buoyancy terms, J/(s m^3^) (for *k*) and kg/(s^2^ m^3^) (for ω).

### 4.1. Geometric Model and Numerical Mesh

The CFD simulations used the same nozzle variants previously manufactured using the FDM technique and tested experimentally, allowing for a direct comparison between the numerical results and real measurements. No simplification of the geometry for CFD was used. Importantly, it is not the solid model itself that is dimensioned but the flow volume, which means that the dimensions retained in Figure 2, Figure 3, Figure 4 and Figure 5 relate directly to the fluid domain and not to the wall thickness of the nozzle as a solid object. Due to the observed breaking phenomena at pressures exceeding 3 bar, the wall thickness of individual nozzles was locally increased to enhance their mechanical strength without compromising the volume of the flow domain. Problems were encountered during experiments with the No. 3 and No. 4 nozzles, particularly when they broke the flow axis. The preserved consistency made it possible to ensure identical boundary conditions during the tests.

Discretization of the computational space was carried out using Ansys Fluent Meshing software (version 2024 R2). Poly-Hexcore meshing elements were used to reduce the number of mesh elements by using different types of elements depending on the layer. This generally reduces the number of elements by 20 to 50% while maintaining the same accuracy [22]. The model inflation at fluid–solid boundaries was applied, and the y+ value was kept at a maximum of less than 1. Due to limitations arising from the use of wall functions, it was decided to represent the viscous near-wall sublayer in the numerical model fully. To assess the quality of its representation, a dimensionless parameter y+ was used, which reflected the relative contribution of viscous and turbulent forces near the wall. Appropriate selection of the value of y+ makes it possible to control the accuracy of flow modeling in this zone. The height of the first cell in the boundary layer was estimated based on empirical relationships describing the development of turbulent flow in the vicinity of the walls [23]:

(8) $$y=\frac{y^{+}\cdot \mu }{\rho \cdot u_{*}}$$

where $y^{+}$—dimensionless wall distance; μ—dynamic viscosity (kg/m s); ρ—density (kg/m^3^); $u_{*}$—friction velocity (m/s).

The skin friction coefficient was calculated using the Schlichting skin friction correlation

(9) $$C_{f}{=[2{log}_{10}\left(Re\right)-0.65]}^{-2.3}$$

where Re = the Reynolds number (-) and was calculated using the formula

(10) $$Re=\frac{\rho \cdot U_{f}\cdot L_{b}}{\mu }$$

where $U_{f}$ = freestream velocity (m/s), and $L_{b}$ = boundary layer length (m).

Friction velocity determines the speed at which momentum is exchanged between the wall and the freestream flow:

(11) $$u_{*}=\sqrt{\frac{\tau_{w}}{\rho }}$$

Determination of the friction velocity requires prior calculation of the wall shear stress, which plays a key role in describing the interaction between the flowing fluid and the wall. Wall shear stress is a measure of the drag forces generated at the wall of a body due to friction, and its value depends on parameters such as the friction coefficient, fluid density, and velocity.

(12) $$\tau_{w}=C_{f}\cdot \frac{1}{2}\rho {U_{f}}^{2}$$

**Figure 9:**
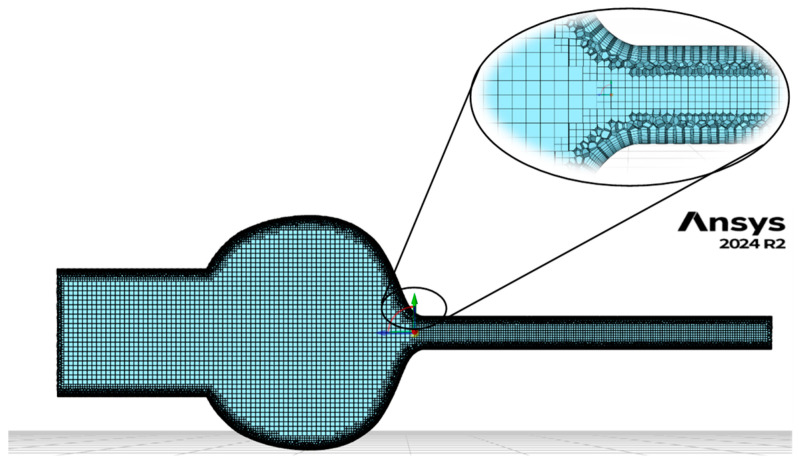
Numerical mesh for nozzle D1.

The height of the first cell from the wall was determined to be about 0.0527 mm in obtaining the assumed value of the y+ number. When determining the thickness of the boundary layer, a criterion was used according to which it was assumed that the flow velocity reached 99% of the maximum value in the core of the stream, which corresponds to the classical definition of the boundary layer thickness *δ*99. This translated into the need to use 8 to 9 layers of inflation, with a coefficient increase in the thickness of successive layers equal to 1.2. An example computational mesh for nozzle geometry No. 1 is presented in Figure 9.

The resulting meshes had 370–450,000 elements, ensuring adequate accuracy and acceptable computation times. The independence of the solution from the number of mesh elements was verified by analyzing the changes in the velocity values at the constriction and the outlet, as well as the mass flux at different numbers of elements. The final mesh was selected so that further densification did not significantly affect the simulation results (less than 0.1%).

### 4.2. Boundary Conditions

**Figure 10:**
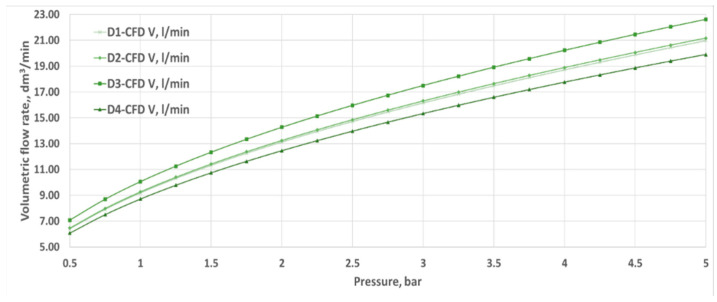
Volumetric flow rates of liquid through the tested water nozzles D1–D4 at different inlet pressures, obtained using the CFD results.

For all nozzle configurations, identical boundary conditions were applied:

- Inlet: A pressure inlet condition defined by the gauge pressure, with tested values of 0.5, 1, 2, 3, 4, and 5 bar (Figure 10);
- Outlet: A pressure outlet condition with a reference overpressure set to 0 Pa;
- Walls: All walls were assumed to be perfectly insulated, i.e., adiabatic, with no heat transfer.

In the numerical analysis, the effect of wall roughness was considered, which is particularly important for parts produced through 3D printing. Based on the experimental data presented in [24], the value of the average surface roughness Ra for PLA was taken as 12.3 µm, which corresponds to printing conditions with a 0.2 mm thick layer. Using this Ra value was found to be more appropriate for CFD modeling purposes than the rate defined in ISO 21920-2:2021 [25] (known as Rz), which describes the average height of the local extremes of the profile. The Rz values reported in the literature (e.g., [26]) for PLA in FDM technology can reach as high as 91 µm, but they are local in nature and do not fully reflect the actual surface hydraulic resistance important to turbulent flow.

The flow was assumed to be incompressible, so a pressure-based solver was employed. The analysis was conducted under steady-state conditions with gravity effects neglected. A coupled scheme was used to solve the governing equations, discretized using second-order methods to ensure higher numerical accuracy. To control solution stability, pseudo-time stepping was disabled, and a Courant-number-based approach was adopted. According to the literature [27], full numerical stability is typically achieved for Courant–Friedrichs–Lewy (CFL) numbers in the range of 5 to 100; in this study, a value of 10 was applied.

## 5. Results of the Water Nozzles Investigations

This section presents a comparison of the experimental and numerical results along the axis of four types of nozzles. The input data for analyzing the nozzle’s operation are summarized in Table 4.

**Figure 11:**
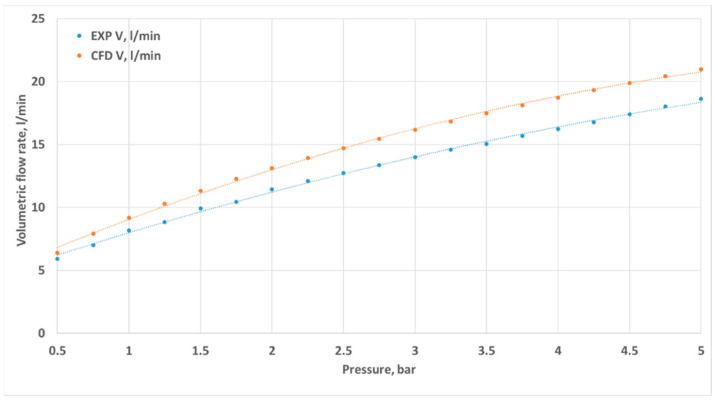
Comparison of the results from experimental and numerical tests for the prototype D1 nozzle in the pressure range of 0.5–5 bar.

**Figure 12:**
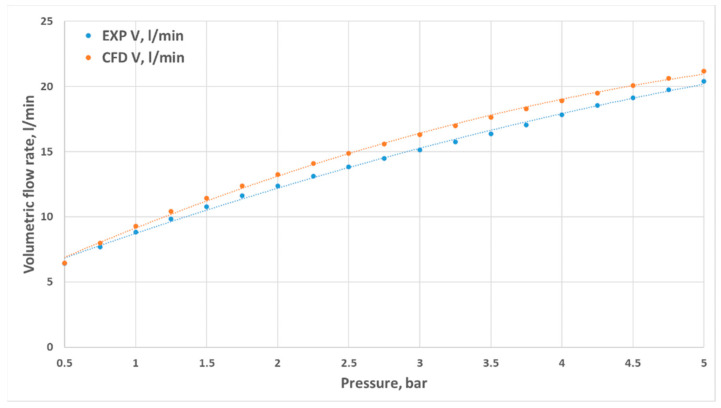
Comparison of the results from experimental and numerical tests for the prototype D2 nozzle in the pressure range of 0.5–5 bar.

**Figure 13:**
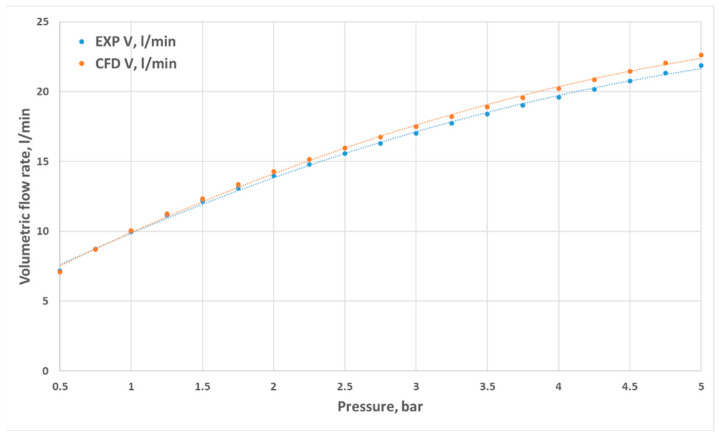
Comparison of the results from experimental and numerical tests for the prototype D3 nozzle in the pressure range of 0.5–5 bar.

**Figure 14:**
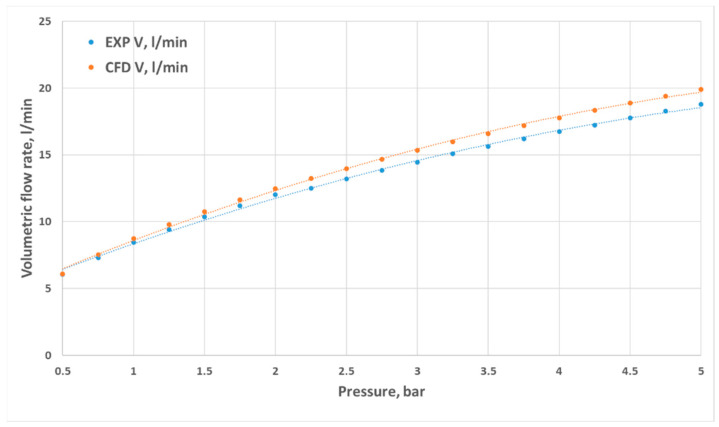
Comparison of the results from experimental and numerical tests for the prototype D4 nozzle in the pressure range of 0.5–5 bar.

**Figure 15:**
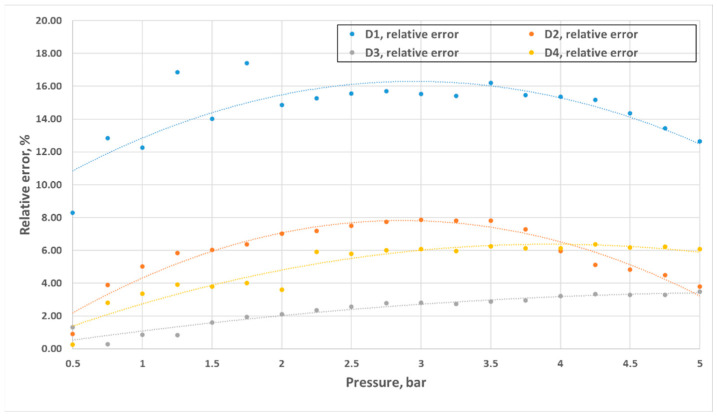
Comparison of the difference values between experimental and numerical results of the prototype D1–D4 nozzles in the pressure range of 0.5–5 bar.

Volumetric flow rates measured through experimental investigation and CFD simulations are presented as a function of inlet pressure in Figure 11, Figure 12, Figure 13 and Figure 14. The inlet pressure and temperature were measured at the nozzle inlet and used as boundary conditions for CFD simulations. During all experimental tests, the water temperature remained within the range of 22.3–24.2 °C. For completeness, the detailed numerical values of the experimental and CFD results, together with the calculated relative errors, are provided in Appendix A Table A1, Table A2, Table A3 and Table A4. The inlet pressure and temperature were measured at the nozzle inlet and used as boundary conditions for CFD simulations. The measured and computed volumetric flow rates, as well as the calculated relative error ε, are presented as a function of the inlet pressure in Figure 11, Figure 12, Figure 13, Figure 14 and Figure 15.

The total difference between the measured and computed values of the volumetric flow rate is lower than 2.55 dm^3^/min (D1) and does not exceed a 17.41% relative error (D1). The most similar results were registered for nozzle No. 3 (D3). The relative errors for nozzles No. 4 and No. 2 (D4, D2) did not exceed the value of 7.87%, and the most significant values were registered for the mid–end of the range of tested pressures (2.5–3.5 bar).

**Figure 16:**
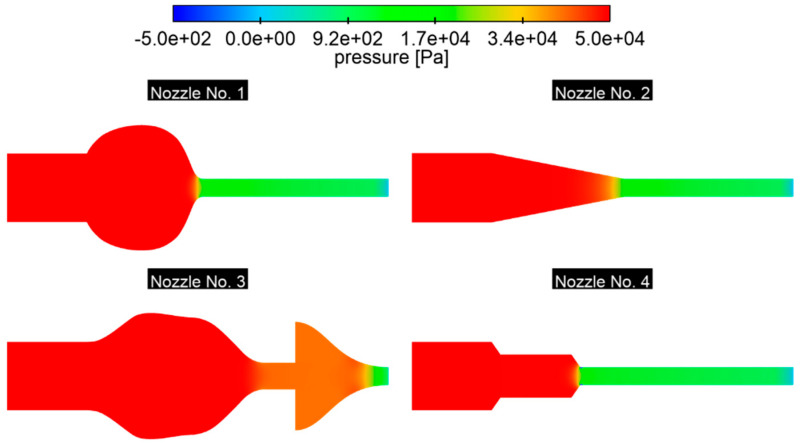
The pressure drop for the longitudinal plane of the nozzle, *p* = 0.5 bar.

Pressure changes in the nozzles are mainly due to cross-sectional variability (Figure 16): in nozzle No. 1, the rapid transition from a spherical chamber to a short connector generates instant pressure drops and sharp dP/dx gradients. In nozzle No. 2, on the other hand, a gently tapering cone extends the pressure drop over the entire length of the element, allowing for almost uniform acceleration of the flow and minimizing energy losses and unfavorable gradients—as a result, the risk of wall layer separation is reduced. Nozzle No. 3 uses two stages of expansion: first, the inlet chamber allows for partial pressure recovery and smoothing of the velocity profile, and then, a gradual diffuser introduces a gradual pressure gradient, strengthening the wall layer before the final steep constriction; as a result, the outlet flow should be more uniform. In nozzle No. 4, sharp edges create an under-pressure zone, which is particularly evident for nozzle No. 4 in the vena contracta area, where kinetic energy completely dominates over pressure energy. This flow pattern creates a risk of cavitation in this area and promotes a suction effect on the walls, which attract fluid–potential structural vibrations. In addition, if the narrowing is too abrupt, recirculation zones may form there, destabilizing the flow. The symmetrical pressure gradient paths suggest that the nozzle’s internal geometry has been properly shaped, promoting stable flow.

**Figure 17:**
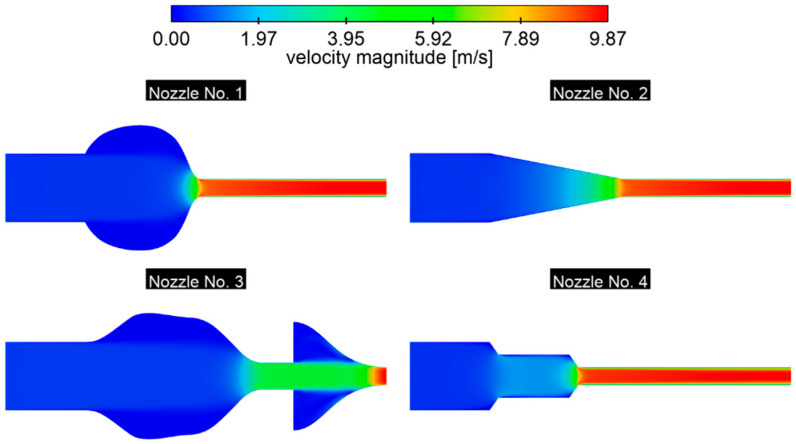
The distribution of the velocity field in the nozzle, *p* = 0.5 bar.

The shape of the channel clearly determines the acceleration dynamics of the stream (Figure 17). Variants with a diffuser, present in nozzles No. 1 and No. 3, do not significantly affect the diameter of the main stream, which remains close to the inlet diameter. On the other hand, sudden constrictions (nozzle 1) generate rapid acceleration, which carries the risk of excessive material stress and a reduced component life. In contrast, the smooth transition in nozzle No. 2 ensures a uniform velocity profile across the entire cross-section, which minimizes the pressure losses. Complex, gradual transitions with a double diffuser (nozzle No. 3) promote velocity irregularities and increase the probability of boundary layer separation. Nozzle No. 4 also appears to be a favorable solution, as a moderate convergence angle and an elongated neck enable stable stretching of the stream core and a reduction in strong gradients. As a result, the main part of the flow dominates the cross-section, and the developed boundary layer in the narrowest section does not disturb the concentration of the maximum velocities on the axis. This phenomenon is not observable for nozzle No. 3, where the boundary layer is not developed.

**Figure 18:**
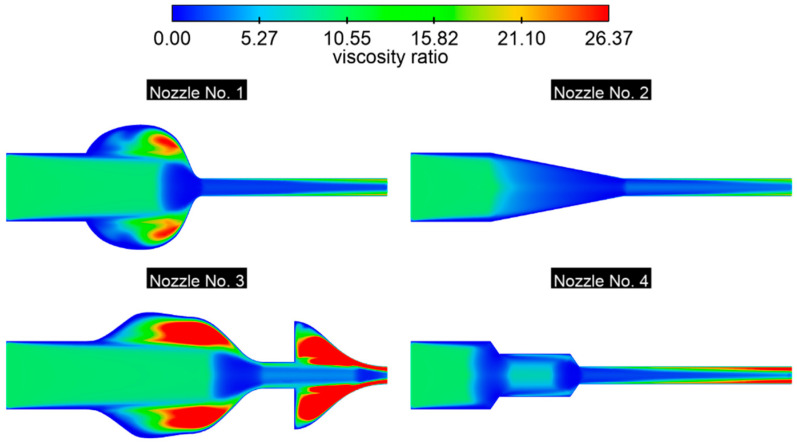
The distribution of the viscosity ratio field in the nozzle, *p* = 0.5 bar.

In general, in order to obtain a full, stable, and energy-efficient flow, it is recommended to design a nozzle with a moderate divergence angle and a gradual profile, as well as an optimal neck length, which allows sudden changes in the cross-section, separation, and excessive development of the boundary layer to be avoided. The turbulent viscosity ratio is defined as μ_t/μ (turbulent-to-molecular-viscosity ratio) and is directly proportional to the turbulent Reynolds number (Figure 18). The higher the value of this coefficient, the slower the turbulence intensity dissipates along the stream. At the inlet, the boundary condition μ_t_/μ = 10 was assumed, but its actual value changed significantly along the flow. The highest values, reaching up to 26, were observed in areas with a diffuser (nozzles No. 1 and No. 3), which corresponds to the recirculation zones in the inlet chamber (Figure 18). The pronounced asymmetry of the distribution suggests the presence of three-dimensional phenomena or a slight disturbance in the axiality of the flow. Identified zones with an increased viscosity ratio may correspond to places where increased energy loss, vortex formation, and turbulence occur, which is important for optimizing nozzle geometry.

Nozzle No. 2 is characterized by low and uniform μ_t_;/μ values along the flow, which decrease as the flow’s cross-section decreases. An increase is only observed in the outlet channel, where the maximum values occur at the walls, but are significantly lower than in other cases. The most pronounced increase was observed in nozzle No. 4, which had the longest neck. This is due to the development of a wall layer, which gradually increases, intensifying the production of turbulent viscosity. In addition, the turbulence model near the wall uses damping functions, initially limiting μ_t_; to zero; then, as y^+^ increases, the turbulent viscosity increases until the value at which turbulence production is in equilibrium with its dissipation. Low turbulent viscosity ratios are most recommended for water flow through nozzles—they guarantee a gradual reduction in turbulence along the channel, reduce friction losses, and minimize flow instabilities. This results in a predictable flow, which is crucial in measurement applications, among others.

**Figure 19:**
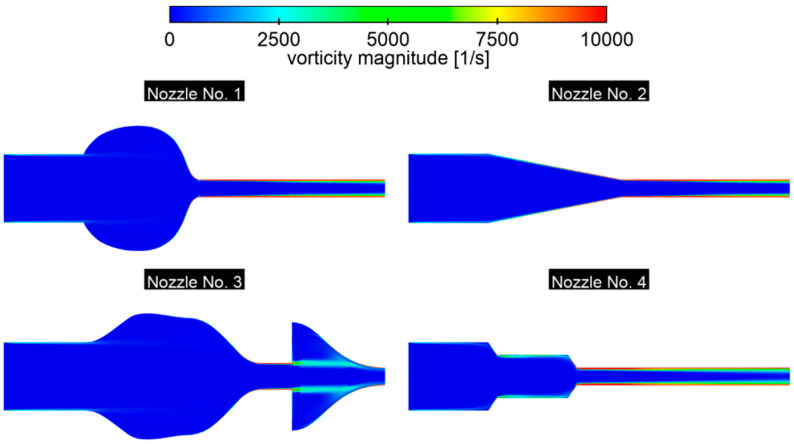
The vorticity magnitude for the longitudinal plane of the nozzle, *p* = 0.5 bar.

An analysis of the vorticity magnitude distribution clearly shows that sudden changes in the cross-section—primarily sharp edges—generate the strongest vortex fields. At such thresholds, vorticity reaches its maximum because the velocity gradient is greatest there. Moreover, in the variants with an extended outlet (especially in nozzle No. 4), the increasingly thick boundary layer favors the formation of moderate, local vortices, which in turn increase friction losses in the flow (Figure 19).

Nozzles No. 1 and 3, equipped with a diffuser in the inlet chamber, exhibit intense mixing and recirculation areas, which translates into high vorticity in the inlet zone. The only variant that ensures uniformly low vorticity values is the gentle convergence in nozzle No. 2—the absence of sudden cross-sectional jumps results in small velocity gradients and a stable, uniform flow.

From the point of view of energy economy and jet controllability, geometries that limit sharp edges and diffusers and promote gradual convergence are most desirable. However, it is worth remembering the compromise: in applications requiring intense mixing, a slight increase in vorticity can be an advantage, improving mass and heat transport.

**Figure 20:**
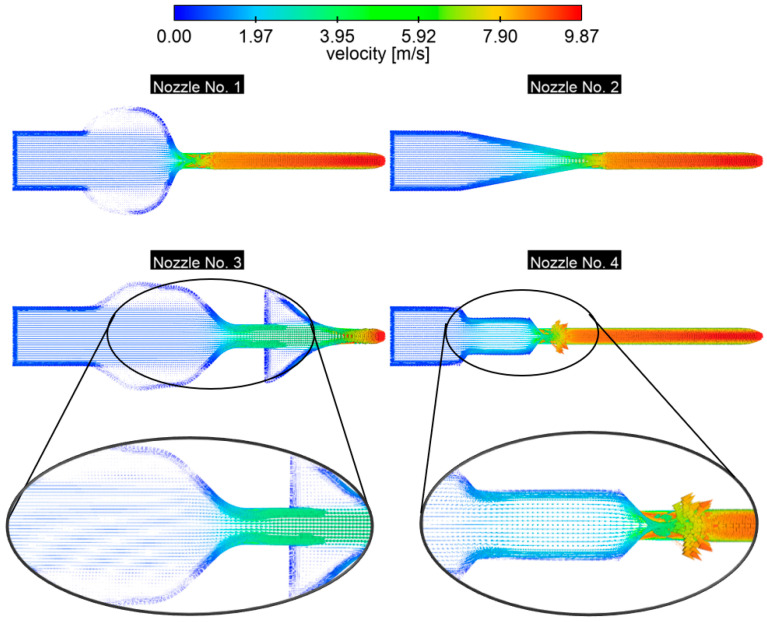
Velocity vectors for the longitudinal plane of the nozzle, *p* = 0.5 bar.

The spacious inlet chamber in nozzle No. 1 generates two symmetrical recirculation bubbles at the walls—the vectors in these zones are directed against the main flow and are short (Figure 20). In the outlet cross-section of the nozzle, the velocity vectors rapidly elongate and straighten, forming a narrow neck core. This contrasting change in vectors (from recirculation vectors to long, axial arrows) determines the locations of the highest shear gradients. For nozzle No. 2, from the inlet to the outlet, we have an almost linear, gradual increase in vector length and a uniform direction. The absence of even slight reflections at the walls indicates practically laminar, stable development of the boundary layer—no local separation bubbles or sharp gradients are formed here. For nozzle No. 3, the enlarged inlet chamber maintains recirculation similar to that in No. 1. The use of a second diffuser results in even larger recirculation zones in the chamber. At the outlet, the vectors are arranged into a core but more irregularly than in nozzle No. 2. No significant recirculation zones were found in nozzle No. 4 despite the presence of gradual narrowing, but at the beginning of the outlet channel, the jet strikes the wall directly, causing rapid local pressure gradients and intensified shear. Such a stream impact carries the risk of damaging the wall surface, causing vibrations and noise, and may also generate jet pulsations and lead to velocity profile asymmetry further into the outlet. To mitigate these negative effects, it is worth considering rounding the convergence edge or introducing small fillets at the neck inlet, which will reduce the impact, limit local hydraulic losses, and improve the uniformity and stability of the flow. A lower boundary layer means a larger effective cross-section of the medium and lower friction resistance in the last section and consequently a higher volume flow.

Geometric differences were directly reflected in the quantitative discharge characteristics. At a 5 bar inlet pressure, the experimental flow rates were 18.63 L/min (D1), 20.40 L/min (D2), 21.86 L/min (D3), and 18.77 L/min (D4) (Appendix A Table A1, Table A2, Table A3 and Table A4). The corresponding experimental flow coefficients were 0.50 m^3^/h (D1), 0.55 m^3^/h (D2), 0.59 m^3^/h (D3), and 0.50 m^3^/h (D4), while the 3D printing efficiency η_3_D reached 88.78%, 96.34%, 96.65%, and 94.28%, respectively (Table 3). The CFD–experiment discrepancies were the largest for D1 (up to 17.41%) and the smallest for D3 (below 3.5%).

The results clearly demonstrate that nozzle geometry, including both diameters and the convergence angle, strongly influences hydraulic performance. The lack of a convergent section (D1) resulted in reduced efficiency and significant deviations between the simulation and the experiment. In contrast, an internal chamber with a convergent section (D3) promoted higher efficiency and improved reproducibility. The elongated throat of D4 stabilized the jet but introduced local under-pressure. Among the tested designs, the staged geometry of D3 proved to be the most advantageous solution, as it combined high discharge efficiency with very good agreement between numerical and experimental results.

## 6. Flow Characteristics of Prototype Water Jets

For the evaluation of individual nozzles, we use coefficients that characterize the efficiency of the liquid outflow through the nozzles, such as the flow coefficient $K_{v}$ determined according to Formula (13). This is an engineering parameter that indicates the amount of liquid that flows through a nozzle at a pressure drop of 1 bar and a temperature of 20 °C. The higher the value $K_{v}$, the greater the flow at the same pressure drop, which can be interpreted as a higher nozzle efficiency.

(13) $$K_{v}=\frac{\dot{V}}{\sqrt{\Delta p}}$$

where $\dot{V}$—volumetric flow rate in the nozzle, m^3^/h; Δp—nozzle pressure drop, bar.

With the results in the form of the fluid flow through the nozzle obtained from the experiment and CFD numerical calculations (Table 5), a $\eta_{3D}$ parameter was proposed to determine the efficiency of 3D printing nozzle fabrication, determined as the quotient of the coefficients $K_{v}$ or the flux quotient for the actual and numerically calculated flow:

(14) $$\eta_{3D}=\frac{K_{vEXP}}{K_{vCFD}}=\frac{{\dot{V}}_{EXP}}{{\dot{V}}_{CFD}}$$

**Figure 21:**
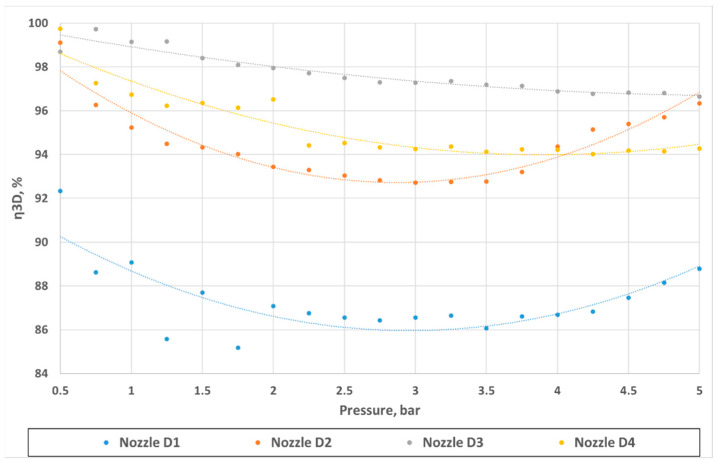
Comparison of nozzle efficiency $\eta_{3D}$ values determined from experimental and numerical data for nozzles D1–D4 as a function of the pressure difference Δ*p*.

The results in Figure 21 show the efficiency of nozzles D1–D4 determined according to the proposed relationship (14). They show differences depending on the inlet pressure, but in each case, the highest values are obtained for the lowest pressures. The lowest efficiency is seen for intermediate pressures, where the greatest discrepancies between the experimental and numerical CFD results also arise. This may indicate the difficulty of modeling the fluid outflow processes through nozzles in the range where the flow is a transient flow, and the behavior of the jet, in particular in the region near the walls and in the flow channel, may require more complex numerical models and methods.

## 7. Conclusions

This study combined steady-state CFD calculations with rapid prototyping through FDM 3D printing to design and assess four water jet nozzles (D1–D4) across a practical inlet pressure range. Experiments conducted on a dedicated test bench and simulations using the developed models captured consistent trends, enabling design-level comparisons. The workflow proved especially reliable for geometries with smooth, gradual convergence while highlighting where additional model fidelity is needed for shapes with sharp area changes.

Across the designs, the best agreement between the measurements and predictions was obtained for D3, followed by D2 and D4, whereas D1 exhibited the largest discrepancies. Flow-physics inspection linked these differences to the internal geometry: gradual acceleration suppressed separation and limited losses; sudden contractions and short connectors promoted recirculation, larger turbulent viscosity ratios, and a higher risk of local under-pressure near the throat (vena-contracta effects). These findings provide clear guidance for shaping the inlet and throat regions to achieve stable, predictable jets. Practically, the results validate using CFD to rank candidate geometries before fabrication and to quantify print-to-design fidelity for polymer nozzles. The combined approach shortens the concept-to-test loop and enables low-cost, iterative optimization of both the geometry and printing parameters. For applications that require steady, well-formed jets, we recommend a smoothly converging profile with a moderated convergence angle and an adequately long throat, together with attention to the surface finish and inlet rounding.

The comparative analysis demonstrated that with the same inlet (15.4 mm), throat/outlet (4 mm), and overall length (86 mm), the internal geometry of the nozzles significantly affected their hydraulic performance. The addition of an intermediate internal chamber further enhanced the discharge capacity, yielding the highest flow rate (21.86 L/min at 5 bar) and the best agreement with CFD (<3.5% error). The elongated throat stabilized the jet but generated local under-pressure. These findings suggest that nozzle optimization cannot rely solely on a single global coefficient but rather requires combined consideration of the geometric parameters, discharge efficiency, and predictive accuracy.

Future extensions should address the mid-range pressure regime, where transitional and cavitation-related effects are most likely and may contribute to residual model–experiment gaps. Targeted refinements—transient simulations with cavitation modeling, systematic variation in the throat length and fillet radii, and explicit characterization of printed surface roughness—are expected to improve the predictive accuracy further and broaden the operational envelope of additively manufactured nozzles.

Future work should also consider the application of statistical methods, such as analysis of variance (ANOVA) [28] or regression analysis, once a larger set of systematically varied nozzle geometries is investigated. This would enable a quantitative assessment of the relative influence of individual geometric parameters on flow performance, providing a more generalizable framework for nozzle optimization.

## Figures and Tables

**Table 1:** Three-dimensional printing parameters for fabricated PLA nozzles.

Printing Settings	Value
Layer height	0.1 mm
Line width	0.4 mm
Wall thickness	1.2 mm
Top/bottom thickness	0.8 mm
Printing speed	30 mm/s
Extruder temperature	210 °C
Build plate temperature	60 °C
Nozzle diameter	0.4 mm
Infill density	100%
Infill patterns	Rectilinear
Fan speed	80%

**Table 2:** The characteristic parameters of the 3D-printed nozzles.

Parameter	D1	D2	D3	D4
Inlet/outlet diameter, mm	15.4/4.0	15.4/4.0	15.4/4.0	15.4/4.0
Total length, mm	86	86	86	86
Number of internal chambers	1	0	2	1
Internal chamber length, mm	26.03	-	14	16
Internal chamber diameter, mm	28.23	-	25.0 24.4	11.7
Convergence angle, °	-	79.1	-	35
Throat length, mm	41.97	38	7	48
Convergent section length, mm	-	40	21	2

**Table 3:** List and main properties of all sensors.

No.	Variable	Range	Sensor	Accuracy
1	Pressure, *p*	−1 ÷ 3 bar 0 ÷ 20 bar	Pressure transducer 0A-10	±0.5%
2	Temperature, *t*	−50 ÷ 180 °C	TOPE-L0384-Pt100-A	±0.15 °C +0.002 × |*t*|
3	Flow rate, $\dot{V}$	0 ÷ 50 dm^3^/min	Flowmeter Picomag, DMA25, DN25 1	±0.1%
4	Current frequency, *f*	0 ÷ 2.2 kW 0 ÷ 60 Hz	Astraada DRV-24 frequencyconverter, 3 × 400 V, vector control, STO.	Speed control accuracy: ±0.2%

**Table 4:** The parameters of the nozzle and water properties adopted for the analysis.

	Nozzle D1	Nozzle D2	Nozzle D3	Nozzle D4
Density, kg/m^3^	9.975 × 10^2^			
Specific volume, m^3^/g	1.000 × 10^−3^			
Dynamic viscosity, Pa·s	1.002 × 10^−3^			
Kinematic viscosity, m^2^/s	1.003 × 10^−6^			
Inlet diameter, m	1.540 × 10^−2^			
Outlet diameter, m	4.000 × 10^−3^			
Inlet area surface, m^2^	1.863 × 10^−1^			
Outlet area surface, m^2^	1.257 × 10^−2^			

**Table 5:** Flow coefficient values determined experimentally $K_{vEXP}$ and numerically $K_{vCFD}$ and the efficiency of prototype manufacturing for nozzles D1–D4 as a function of the pressure drop.

	Nozzle D1				Nozzle D2				Nozzle D3				Nozzle D4			
Δ*p*	*Kv_EXP_*	*Kv_CFD_*	Δ*V*	*η_3D_*	*Kv_exp_*	*Kv_CFD_*	Δ*V*	*η_3D_*	*Kv_exp_*	*Kv_CFD_*	Δ*V*	*η_3D_*	*Kv_exp_*	*Kv_CFD_*	Δ*V*	*η_3D_*
bar	m^3^/h	m^3^/h	dm^3^/min	%	m^3^/h	m^3^/h	dm^3^/min	%	m^3^/h	m^3^/h	dm^3^/min	%	m^3^/h	m^3^/h	dm^3^/min	%
0.50	0.50	0.54	0.49	92.34	0.54	0.55	0.06	99.11	0.61	0.60	0.09	98.69	0.52	0.52	0.02	99.75
0.75	0.49	0.55	0.90	88.62	0.53	0.55	0.30	96.26	0.60	0.60	0.02	99.72	0.51	0.52	0.21	97.26
1.00	0.49	0.55	1.00	89.08	0.53	0.56	0.44	95.23	0.60	0.60	0.09	99.15	0.51	0.52	0.28	96.74
1.25	0.47	0.55	1.49	85.58	0.53	0.56	0.57	94.48	0.60	0.60	0.09	99.17	0.51	0.53	0.37	96.23
1.50	0.49	0.55	1.39	87.70	0.53	0.56	0.65	94.32	0.59	0.60	0.20	98.41	0.51	0.53	0.39	96.35
1.75	0.47	0.56	1.82	85.17	0.53	0.56	0.74	94.01	0.59	0.60	0.25	98.10	0.51	0.53	0.45	96.14
2.00	0.48	0.56	1.70	87.07	0.53	0.56	0.87	93.44	0.59	0.61	0.29	97.94	0.51	0.53	0.43	96.51
2.25	0.48	0.56	1.85	86.76	0.53	0.56	0.94	93.29	0.59	0.61	0.35	97.71	0.50	0.53	0.74	94.41
2.50	0.48	0.56	1.98	86.55	0.52	0.56	1.04	93.03	0.59	0.61	0.40	97.49	0.50	0.53	0.76	94.53
2.75	0.48	0.56	2.10	86.43	0.52	0.56	1.12	92.83	0.59	0.61	0.45	97.29	0.50	0.53	0.83	94.33
3.00	0.48	0.56	2.17	86.56	0.52	0.56	1.19	92.71	0.59	0.61	0.48	97.27	0.50	0.53	0.88	94.26
3.25	0.49	0.56	2.25	86.65	0.52	0.57	1.23	92.75	0.59	0.61	0.48	97.35	0.50	0.53	0.90	94.37
3.50	0.48	0.56	2.44	86.06	0.53	0.57	1.28	92.76	0.59	0.61	0.53	97.19	0.50	0.53	0.98	94.12
3.75	0.49	0.56	2.43	86.61	0.53	0.57	1.24	93.21	0.59	0.61	0.56	97.13	0.50	0.53	0.99	94.23
4.00	0.49	0.56	2.49	86.68	0.53	0.57	1.06	94.37	0.59	0.61	0.63	96.88	0.50	0.53	1.03	94.22
4.25	0.49	0.56	2.54	86.83	0.54	0.57	0.95	95.14	0.59	0.61	0.67	96.77	0.50	0.53	1.10	94.02
4.50	0.49	0.56	2.50	87.45	0.54	0.57	0.93	95.39	0.59	0.61	0.68	96.82	0.50	0.53	1.10	94.18
4.75	0.50	0.56	2.42	88.15	0.54	0.57	0.89	95.70	0.59	0.61	0.70	96.80	0.50	0.53	1.13	94.15
5.00	0.50	0.56	2.35	88.78	0.55	0.57	0.77	96.34	0.59	0.61	0.76	96.65	0.50	0.53	1.14	94.28

## Data Availability

The original contributions presented in this study are included in the article. Further inquiries can be directed to the corresponding author.

## Appendix A

**Table A1:** Results for the D1 nozzle obtained in the experiment and numerical studies using CFD.

Inlet Pressure *p_in_*, Bar	Inlet Temperature *t_in_*, °C	$\mathrm{Measured}\;\mathrm{Flow}\;\mathrm{Rate}\;{\dot{V}}_{EXP}$, dm^3^/min	$\mathrm{Calculated}\;\mathrm{Flow}\;\mathrm{Rate}\;{\dot{V}}_{CFD}$, dm^3^/min	$\mathrm{Difference}\;\Delta \dot{V}$, dm^3^/min	Relative Error *ε*, %
0.50	23.26	5.91	6.40	0.49	8.30
0.75	23.27	7.01	7.91	0.90	12.84
1.00	23.27	8.18	9.18	1.00	12.26
1.25	23.27	8.82	10.31	1.49	16.85
1.50	23.27	9.93	11.32	1.39	14.02
1.75	23.27	10.44	12.26	1.82	17.41
2.00	23.27	11.43	13.13	1.70	14.85
2.25	23.43	12.10	13.95	1.85	15.26
2.50	23.48	12.74	14.72	1.98	15.54
2.75	23.69	13.36	15.46	2.10	15.70
3.00	23.77	13.99	16.16	2.17	15.52
3.25	23.92	14.59	16.84	2.25	15.40
3.50	23.94	15.05	17.49	2.44	16.20
3.75	24.15	15.69	18.12	2.43	15.46
4.00	24.15	16.23	18.72	2.49	15.36
4.25	24.08	16.77	19.31	2.54	15.16
4.50	24.15	17.39	19.89	2.50	14.35
4.75	24.16	18.02	20.44	2.42	13.44
5.00	24.20	18.63	20.98	2.35	12.64

**Table A2:** Results for the D2 nozzle obtained from the experiment and numerical studies using CFD.

Inlet Pressure *p_in_*, Bar	Inlet Temperature *t_in_*, °C	$\mathrm{Measured}\;\mathrm{Flow}\;\mathrm{Rate}\;{\dot{V}}_{EXP}$, dm^3^/min	$\mathrm{Calculated}\;\mathrm{Flow}\;\mathrm{Rate}\;{\dot{V}}_{CFD}$, dm^3^/min	$\mathrm{Difference}\;\Delta \dot{V}$, dm^3^/min	Relative Error *ε*, %
0.50	23.48	6.41	6.47	0.06	0.90
0.75	23.52	7.69	7.99	0.30	3.88
1.00	23.48	8.83	9.27	0.44	5.01
1.25	23.48	9.83	10.40	0.57	5.84
1.50	23.48	10.78	11.43	0.65	6.02
1.75	23.48	11.63	12.37	0.74	6.38
2.00	23.43	12.38	13.25	0.87	7.02
2.25	23.49	13.13	14.07	0.94	7.20
2.50	23.43	13.82	14.86	1.04	7.49
2.75	23.43	14.48	15.60	1.12	7.73
3.00	23.48	15.12	16.31	1.19	7.87
3.25	23.56	15.76	16.99	1.23	7.81
3.50	23.43	16.37	17.65	1.28	7.80
3.75	23.43	17.04	18.28	1.24	7.28
4.00	23.43	17.83	18.89	1.06	5.97
4.25	23.43	18.54	19.49	0.95	5.11
4.50	23.43	19.14	20.07	0.93	4.83
4.75	23.43	19.74	20.63	0.89	4.49
5.00	23.43	20.40	21.17	0.77	3.79

**Table A3:** Results for the D3 nozzle obtained from the experiment and numerical studies using CFD.

Inlet Pressure *p_in_*, Bar	Inlet Temperature *t_in_*, °C	$\mathrm{Measured}\;\mathrm{Flow}\;\mathrm{Rate}\;{\dot{V}}_{EXP}$, dm^3^/min	$\mathrm{Calculated}\;\mathrm{Flow}\;\mathrm{Rate}\;{\dot{V}}_{CFD}$, dm^3^/min	$\mathrm{Difference}\;\Delta \dot{V}$, dm^3^/min	Relative Error *ε*, %
0.50	23.27	7.17	7.08	−0.09	1.31
0.75	23.36	8.72	8.70	-−0.02	0.28
1.00	23.36	9.97	10.06	0.09	0.85
1.25	23.27	11.16	11.25	0.09	0.83
1.50	23.36	12.14	12.34	0.20	1.62
1.75	23.27	13.08	13.33	0.25	1.94
2.00	23.36	13.97	14.26	0.29	2.10
2.25	23.43	14.79	15.14	0.35	2.34
2.50	23.43	15.56	15.96	0.40	2.58
2.75	23.48	16.29	16.74	0.45	2.78
3.00	23.60	17.02	17.50	0.48	2.81
3.25	23.60	17.73	18.21	0.48	2.73
3.50	23.43	18.38	18.91	0.53	2.89
3.75	23.43	19.01	19.57	0.56	2.96
4.00	23.36	19.59	20.22	0.63	3.22
4.25	23.36	20.17	20.84	0.67	3.34
4.50	23.36	20.77	21.45	0.68	3.29
4.75	23.36	21.34	22.04	0.70	3.30
5.00	23.61	21.86	22.62	0.76	3.47

**Table A4:** Results for the D4 nozzle obtained from the experiment and numerical studies using CFD.

Inlet Pressure *p_in_*, Bar	Inlet Temperature *t_in_*, °C	$\mathrm{Measured}\;\mathrm{Flow}\;\mathrm{Rate}\;{\dot{V}}_{EXP}$, dm^3^/min	$\mathrm{Calculated}\;\mathrm{Flow}\;\mathrm{Rate}\;{\dot{V}}_{CFD}$, dm^3^/min	$\mathrm{Difference}\;\Delta \dot{V}$, dm^3^/min	Relative Error *ε*, %
0.50	23.68	6.07	6.09	0.02	0.25
0.75	23.76	7.31	7.52	0.21	2.82
1.00	23.81	8.44	8.72	0.28	3.36
1.25	23.81	9.42	9.79	0.37	3.92
1.50	23.81	10.36	10.75	0.39	3.79
1.75	23.81	11.19	11.64	0.45	4.01
2.00	23.81	12.03	12.46	0.43	3.61
2.25	23.01	12.50	13.24	0.74	5.92
2.50	23.13	13.21	13.97	0.76	5.78
2.75	23.25	13.84	14.67	0.83	6.01
3.00	23.25	14.46	15.34	0.88	6.08
3.25	23.01	15.08	15.98	0.90	5.97
3.50	23.01	15.62	16.60	0.98	6.25
3.75	23.01	16.2	17.19	0.99	6.12
4.00	23.14	16.74	17.77	1.03	6.14
4.25	23.02	17.23	18.33	1.10	6.36
4.50	22.71	17.77	18.87	1.10	6.18
4.75	22.58	18.26	19.39	1.13	6.22
5.00	22.30	18.77	19.91	1.14	6.07
